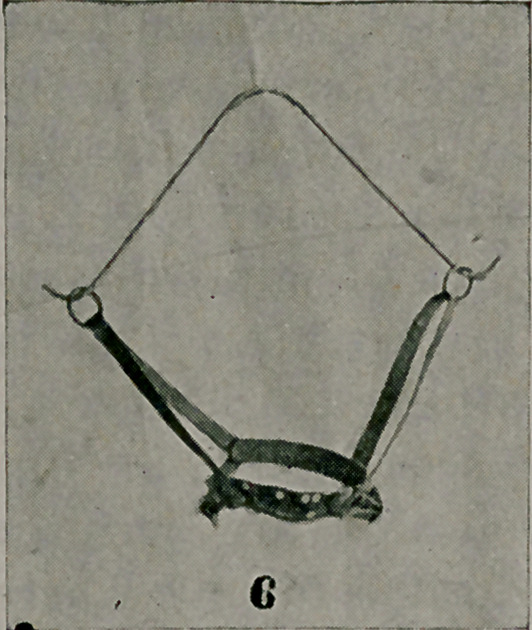# Various Orthopedic Appliances

**Published:** 1903-12

**Authors:** Prescott Le Breton

**Affiliations:** Assistant attending orthopedic surgeon, Children’s and Erie County Hospitals, Buffalo.


### Various Orthopedic Appliances.

By PRESCOTT LE BRETON, M. D.,

Assistant attending orthopedic surgeon, Children's and Erie County Hospitals, Buffalo.

IN photograph No. 1, may be seen a corrective machine for rigid cases of flat foot. The machine was devised by Dr. Robert W. Lovett, of Boston, and the writer obtained one through his courtesy. It is a simple contrivance, so arranged that while the anterior arch of the foot and the heel are held securely in one position, a broad band of strong webbing encircling the tarsus is tightened gradually by screw power. The direction of the force applied is upward and outward, thus restoring the arch of the foot and inverting the forefoot. The head of the astragalus is drawn upward and outward to its normal position. This machine is naturally of service, only when rigidity is present. The use of a plate or raised insole is always contraindicated until the deformity can be passively corrected. Great rigidity and numerous adhesions call for forcible correction under an anesthetic as heretofore, but the after-treatment is facilitated by the use of this machine. The foot is held firmly in the corrected position for ten to fifteen minutes daily, until normal motion is assured. Active and passive movements supplement this treatment and later the arch can be supported.

In the spine and in the foot we find analogous conditions. Numerous small bones and ligaments are concerned in each movement. Just as for the rigid lateral curvatures increased freedom of motion may be afforded by the action of the screw pressure machine, so in flatfoot this machine will accomplish the desired end and do this more effectually than passive correction with the hands alone.

In the other photographs are illustrations of some uses of leather for apparatus besides the conventional leather jacket. The principle of construction is the same no matter for what part of the body the appliance is intended. A plaster-of-paris form is obtained by wrapping the part in bandages and removing the shell immediately. This form is filled with soft plaster and then removed as soon as the soft plaster sets. Correction of the cast follows by a remodeling process if desired. In other words, the plaster is cut away in places and soft plaster added to corresponding portions until symmetry is secured. The cast is wrapped in a light layer of bandage to prevent it from cracking, and all wrinkles smoothed by light tapping with a hammer. A piece of wet leather of suitable size and with beveled edges is wrapped about the cast and wound firmly with window cord or rope until the leather hugs the cast closely. In a few days drying is complete and the leather may be removed and trimmed. It is perforated in many places, sandpapered, and given at intervals several coats of shellac. Bands of webbing with buckles are riveted on at convenient intervals and the apparatus is finished. The inner surface may be lined with chamois, for example, the neck portion of the leather seen in Fig. 2. Portions of the leather corresponding to flexures of joints may need strengthening, also other areas exposed to pressure. This is accomplished by gluing and riveting extra pieces of leather to the original, *e. g.*, the inner side of the shoe seen in Fig. 5. In general it may be said that fixation and protection are furnished by such apparatus, therefore they are indicated mainly where acute symptoms have subsided and extension is not desirable. They are excellent also as night splints, preventing relapse of deformity during the sleeping hours of the patients.

A combination collar and half-jacket is represented in Fig. 2, which is satisfactory in a case of Pott's disease of the cervical region after the inflammatory process has ceased. The leather overlaps at the back and the armholes allow free motion for the arms. The upper part of the trunk, the neck, the chin and the occiput are thus held firmly, but not rigidly. The skin of the patient is separated from the leather by the chamois lining above, and a closefitting undershirt below. A large necktie will cover up the greater part of the leather above.

A hip spica is shown in the next photograph. This is as useful as the familiar Thomas splint. A high shoe on the opposite foot and the use of crutches are necessary until the patient is convalescent. If extension is also desired a skeleton traction splint may be attached to the leather about the waist and to the shoe below, as advocated by Lovett (*New York Medical Journal,* August 24, 1901).

The long leg splint in Fig. 4, is of service as a night appli-

1

2

3

4

6

5

ance to prevent recurrence of deformity after straightening at the knee-joint, *e. g.*, after a transplantation of contractured hamstring tendons to a paralysed quadriceps extensor. The short support was made for use at night for a case of tubercular knee-joint, a Thomas extension brace being worn in the daytime.

The clubfoot shoes in the photograph (Fig. 5) are also exceedingly useful for night use. Given a case of mild clubfoot where the deformity can be passively corrected by the hands, or a severe case which has been operated upon, the feet will remain in the old deformed position during sleep without some corrective appliance. Leather shoes of this type are comfortable and continue the corrective force of whatever apparatus is used during the day. The plaster cast has to be remodeled and the leather to the inner side of the metatarsus strengthened. In wrapping the cast the leather bunches at the outer malleolus and a V-shaped piece must be removed and the remaining edges united by a tongue of soft leather beneath. The webbing is applied in such a way that on buckling it the foot is everted still more.

The picture (Fig. 6) depicts a very simple imitation of the Sayre suspension apparatus. As this appliance is rather expensive and as numerous cases of spinal affections appear in the hospitals and in private practice where traction on the head in bed is indicated, the writer decided to make several of these. For a few cents the necessary rings, buckles and steel shafting were purchased. Over a plaster cast of a chin was modeled the chin piece. Remnants of leather which had been used for jackets were shaved to a proper degree of thickness at a leather establishment. From these the straps and small pieces were cut, and then soaped to render them pliable. Very little time was expended in fastening them together. One-quarter-inch steel shafting was cut to the proper length and bent as seen in the photograph. In this way can be easily obtained a copy of the Sayre's apparatus which is simple, light and capable of sustaining a continued pull of fifteen or twenty pounds.

20 Carlton Street.

## Figures and Tables

**Figure f1:**
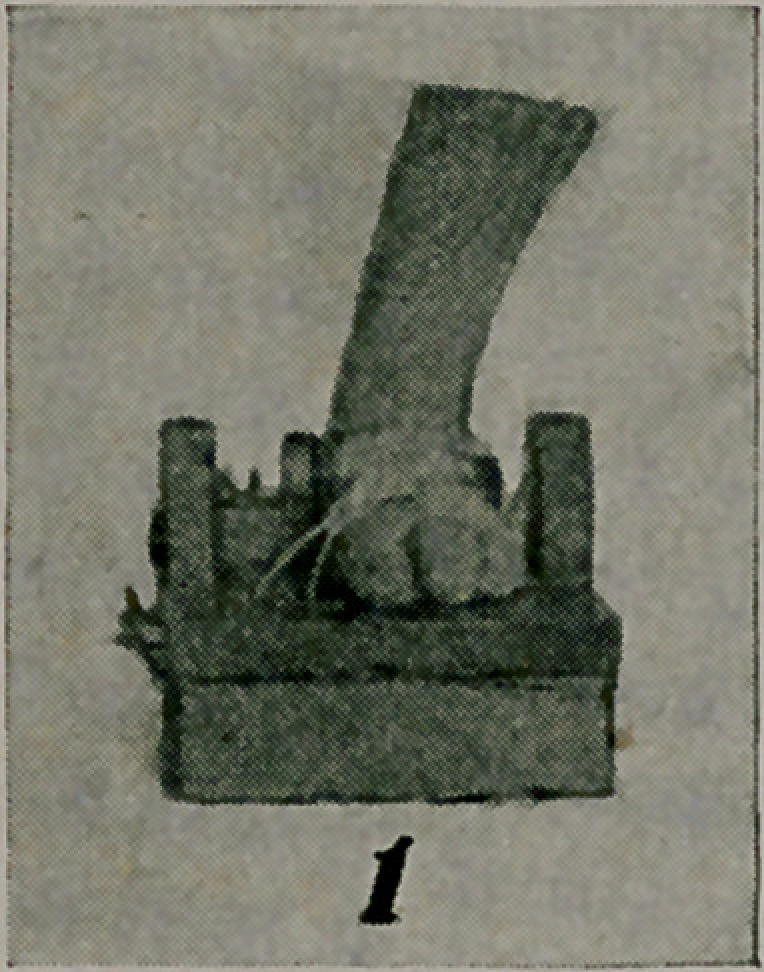


**Figure f2:**
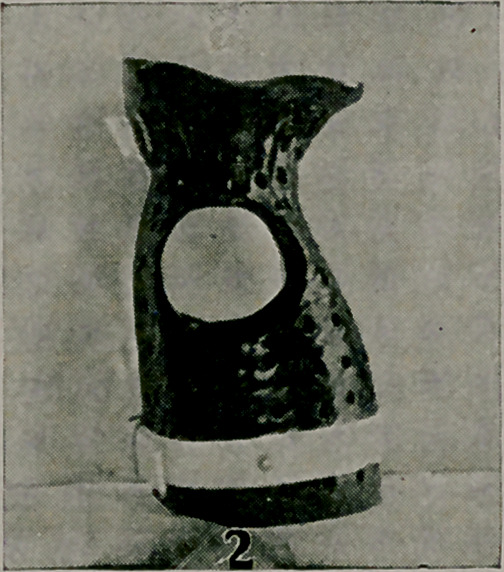


**Figure f3:**
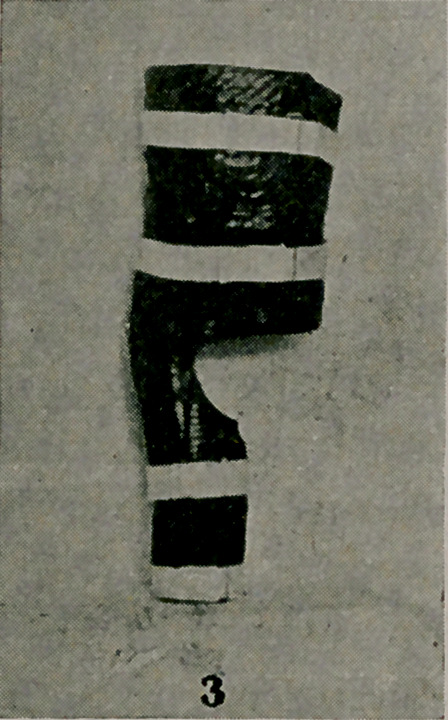


**Figure f4:**
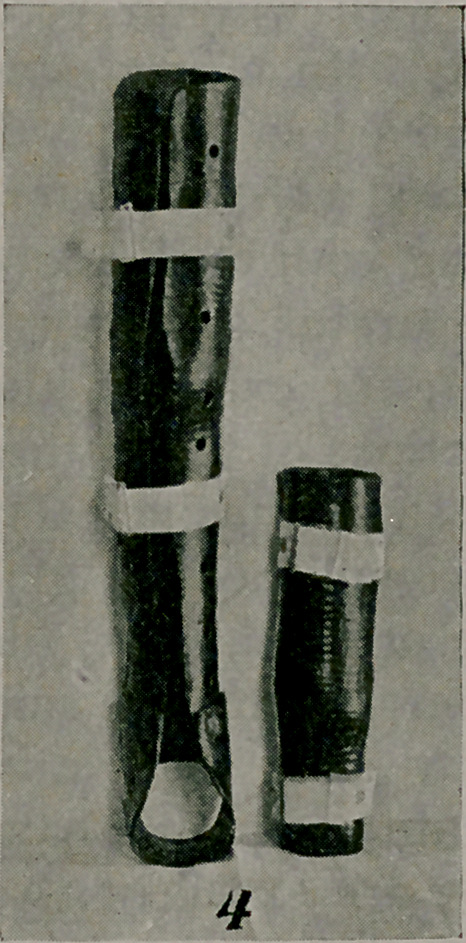


**Figure f5:**
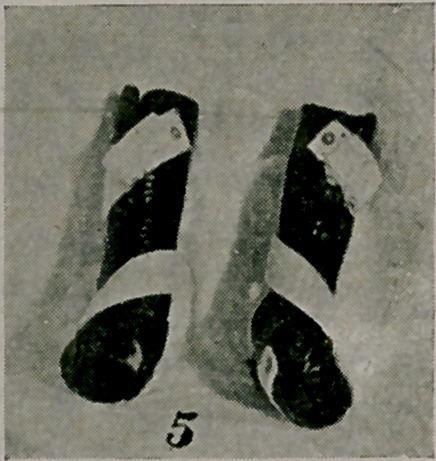


**Figure f6:**